# Antifungal activity of Fe_3_O_4_@SiO_2_/Schiff-base/Cu(II) magnetic nanoparticles against pathogenic Candida species

**DOI:** 10.1038/s41598-024-56512-5

**Published:** 2024-03-11

**Authors:** Sedigheh Azadi, Esmat Azizipour, Ali Mohammad Amani, Ahmad Vaez, Zahra Zareshahrabadi, Alireza Abbaspour, Tahereh Firuzyar, Hengameh Dortaj, Hesam Kamyab, Shreeshivadasan Chelliapan, Sareh Mosleh-Shirazi

**Affiliations:** 1 Department of Medical Nanotechnology, School of Advanced Medical Sciences and Technologies, Shiraz University of Medical Sciences, Shiraz, Iran; 2 Department of Tissue Engineering and Applied Cell Sciences, Shiraz University of Medical Sciences, Shiraz, Iran; 3 Basic Sciences in Infectious Diseases Research Center, Shiraz University of Medical Sciences, Shiraz, Iran; 4 School of Medicine, Shiraz University of Medical Sciences, Shiraz, Iran; 5 Department of Nuclear Medicine, School of Medicine, Shiraz University of Medical Sciences, Shiraz, Iran; 6 Department of Anatomy and Cell Biology, Mashhad University of Medical Sciences, Mashhad, Iran; 7 Faculty of Architecture and Urbanism, UTE University, Calle Rumipamba S/N and Bourgeois, Quito, Ecuador; 8 Department of Biomaterials, Saveetha Dental College and Hospital, Saveetha Institute of Medical and Technical Sciences, Chennai, 600077 India; 9 Process Systems Engineering Centre (PROSPECT), Faculty of Chemical and Energy Engineering, Faculty of Engineering, Universiti Teknologi Malaysia, Skudai, Johor Malaysia; 10 Department of Engineering and Technology, Razak Faculty of Technology and Informatics, Universiti Teknologi Malaysia, Jalan Sultan Yahya Petra, 54100 Kuala Lumpur, Malaysia; 11 Department of Materials Science and Engineering, Shiraz University of Technology, Shiraz, Iran

**Keywords:** Cu(II) Nanoparticles, Candida species, Anti-fungal property, Microdilution, Cytotoxicity, Fungal pathogenesis, Synthetic chemistry methodology, Pathogenesis, Chemistry

## Abstract

The antifungal efficacy and cytotoxicity of a novel nano-antifungal agent, the Fe_3_O_4_@SiO_2_/Schiff-base complex of Cu(II) magnetic nanoparticles (MNPs), have been assessed for targeting drug-resistant Candida species. Due to the rising issue of fungal infections, especially candidiasis, and resistance to traditional antifungals, there is an urgent need for new therapeutic strategies. Utilizing Schiff-base ligands known for their broad-spectrum antimicrobial activity, the Fe_3_O_4_@SiO_2_/Schiff-base/Cu(II) MNPs have been synthesized. The Fe_3_O_4_@SiO_2_/Schiff-base/Cu(II) MNPs was characterized by Fourier Transform-Infrared Spectroscopy (FT-IR), X-ray Diffraction (XRD), Transmission Electron Microscopy (TEM), Scanning Electron Microscopy (SEM), Dynamic Light Scattering (DLS), Energy-dispersive X-ray (EDX), Vibrating Sample Magnetometer (VSM), and Thermogravimetric analysis (TGA), demonstrating successful synthesis. The antifungal potential was evaluated against six Candida species (*C*. *dubliniensis*, *C*. *krusei*, *C*. *tropicalis*, *C*. *parapsilosis*, *C*. *glabrata*, and *C*. *albicans*) using the broth microdilution method. The results indicated strong antifungal activity in the range of 8–64 μg/mL with the lowest MIC (8 μg/mL) observed against *C*. *parapsilosis*. The result showed the MIC of 32 μg/mL against *C. albicans* as the most common infection source. The antifungal mechanism is likely due to the disruption of the fungal cell wall and membrane, along with increased reactive oxygen species (ROS) generation leading to cell death. The MTT (3-[4,5-dimethylthiazol-2-yl]-2,5-diphenyltetrazolium bromide) assay for cytotoxicity on mouse L929 fibroblastic cells suggested low toxicity and even enhanced cell proliferation at certain concentrations. This study demonstrates the promise of Fe_3_O_4_@SiO_2_/Schiff-base/Cu(II) MNPs as a potent antifungal agent with potential applications in the treatment of life-threatening fungal infections, healthcare-associated infections, and beyond.

## Introduction

Fungi infections have become a serious health danger to humans worldwide being difficult to deal with due to the ever-increasing level of non-immunocompromised conditions which leads to invasive infections and high mortality^1,2^. Among the various fungal infections that exist, *Candida* species are prevalent and possess considerable influence on human health^3^, causing severe infections name as candidiasis, and ultimately death in susceptible patients^4^. Based on previous research, *C. albicans* has occupied the first position (80%) of the *Candida* infections^4^. Moreover, the number of infections has increased by *C*. *tropicalis*, *C*. *krusei* and *C*. *parapsilosis*^5^.

Various types of antifungal therapeutic agents including alkylamines, azoles, polyenes, echinocandins, and 5-fluorocytosine have been applied in the eradication of *Candida* infections^4^. Fluconazole, voriconazole, and amphotericin B (AmpB) are important antifungals used against these infections^6^. However, the development of pathogenic *Candida* species resistance and failure in the conventional treatment have set the stage for novel and alternative antifungals to prevent drug persistence and mortality rate^6,7^. By developing the antifungals, the Schiff-base complex of metal ions performs a substantial role in the development of biochemistry, pharmacy, and medicine, which exhibits potential abilities as antifungal, antibacterial, anticancer, anti-inflammatory, antitumor, and antiviral^8–10^. As the metal ions share their partial charge with the donor groups, the polarity of metal ions reduces while the lipophilicity increases, so the lipid layer of microorganisms is more susceptible to these coordinated metals, which leads to massive destruction^11^.

The antifungal activity of various Schiff-base complexes of metal ions like Cu^12^, Co^13^, Zn^14^, Fe^15^, Mn^8^ have been studied, among which Cu coordinated with Schiff-base ligands is a new potential antifungal agent to expand the explorations. The following reports are on the antifungal ability of Cu Schiff-base complex of 2-((E)-(2–hydroxyethylimino)methyl)-1-naptaphenol^9^, Cu(II) complexes of porphyrin core^13^, thiophene-derived Schiff base complex of Cu (II)^16^, antipyrine derived-Schiff base Cu complex^17^, Cu(II) complexes with terpene derivatives of ethylenediamine^18^, Cu(II) complex containing NO donor bidentate Schiff base^19^, thiazole Schiff base complex of Cu^20^, 4,6-dihydroxy-1,3-phenylenediethanone Schiff base complex of Cu derived from^21^, and Cu(II) complex of Schiff-base based on glycine and phenylalanine^19^.

The emergence of nanoscience and nanotechnology as a multidisciplinary area paves the way for investigating the antimicrobial properties of metallic nanoparticles NPs^22^. The enhanced biological activities of NPs result from the decrease in the size, the increase in the surface-to-volume ratio of particles, and less toxicity, which sets the stage for strong interaction with the membranes of microorganisms^36^. Various nanomaterials have been recently reported as antifungal agents like biogenic silver NPs^23^, MNPs bearing metallocarbonyl moiety^24^, MoSe_2_/chitosan nanosheets^25^, curcumin loaded chitosan NPs^26^, and chitosan mediated gold NPs^27^.

Among nanomaterials, metallic NPs have been applied in different fields like engineering photovoltaic technology, electronic, information storage, catalytic, chemical, environmental technology, biosensors, medicine, and biomedical fields^3,28^. Moreover, metal NPs are potential candidates to prevent fungal proliferation in foodstuffs and be used as antifungal agents as well^29^. Among the metallic NPs, Cu NPs have demonstrated enhanced fungicidal and antifungal capability for a wide range of fungi species^30^. For example, Cu NPs capped in starch and sodium alginate represented antifungal activity against *C. albicans* and *C. krusei* fungi.^3^, and Fe_3_O_4_@PDA/Cu(II) NPs showed antifungal properties against *C. guilliermondii*, *C. parapsilosis*, *C. albicans*, *C. krusei*, and *C. glabrata* fungi^31^.

The Schiff-base complex of metal NPs can be immobilized on the surface of Fe_3_O_4_@SiO_2_ core–shell MNPs, which results in more air and thermal stability^32,33^. The immobilization of the Fe_3_O_4_@SiO_2_ MNPs not only results in more air and thermal stability but also increases efficiency due to the high surface-to-volume ratio^33^. The Fe_3_O_4_@SiO_2_/Schiff-base complex of Cu has exhibited remarkable catalytic abilities in organic reactions like *N*-arylation of imidazole with aryl halides^34^, selective mono *N*-arylation of primary *O*-alkyl thiocarbamates and primary *O*-alkyl carbamates with aryl halides and aryl boronic acids^35^, the synthesis of 2-amino-*4H*-chromene derivatives^36^, and oxidation of olefins^37^.

In light of the ever-increasing significance of developing antifungals and nano-sized materials, making antifungals based on nano-scale paves the way for facing fungal infections more properly. Following the excellent catalytic ability of Fe_3_O_4_@SiO_2_/Schiff-base/Cu MNPs, we turn to evaluate its biological activity and broaden the scope of the Cu NPs. The purpose of the present study is to prepare, characterize, and explore the antifungal ability of Fe_3_O_4_@SiO_2_/Schiff-base complex of Cu(II) NPs against six *Candida* species using the microdilution method and study its cytotoxicity by MTT test. This research has gained acceptance from the Ethics Committee of Shiraz University of Medical Sciences: http://ethics.research.ac.ir/IR.SUMS.REC.1402.044.

## Materials and methods

The materials for the synthesis of Fe_3_O_4_@SiO_2_/Schiff-base/Cu(II) MNPs, iron (III) chloride.6H_2_O (FeCl_3_.6H_2_O), iron (II) chloride.4H_2_O (FeCl_2_.4H_2_O), cetyltrimethylammonium bromide (CTAB), sodium hydroxide (NaOH), Tetraethyl orthosilicate (TEOS), 2-hydroxy benzaldehyde (salicylaldehyde), 3-aminopropyltriethoxysilane (APTES), copper(II) acetate.4H_2_O [Cu(OAc)_2_.4H_2_O], were purchased from Sigma-Aldrich. The MTT material for the cell viability was bought from Merck. Water (H_2_O), ethanol (EtOH), and dimethyl sulfoxide (DMSO) were purchased from Merck and applied with no more purification.

The quantitative and qualitative analytical methods were used to characterize and confirm the successful synthesis of the Fe_3_O_4_@SiO_2_/Schiff-base/Cu(II). The FT-IR spectra of the materials were recorded on a Tensor II spectrophotometer using the potassium bromide (KBr) pellet at 400–4000 cm^−1^ (Brucker company). XRD analysis was used to investigate the phase composition and the crystalline structure of MNPs by Bruker AXS D8-Advance X-ray diffractometer with Cu Kα radiation (λ = 1.5418) for 2θ values over the range of 10–80. EDX was utilized to characterize the elements of materials. VSM technique was applied to measure the magnetization of NPs by a BHV-55 VSM using a magnetic field up to 8000 Oe at 300 K. TGA determined the thermal stability of the NPs using a NETZSCH STA 409 PC/PG in the temperature range of 25–750 °C with a heating rate of 10 ˚C min^-1^ under a nitrogen atmosphere. The TEM image was recorded by a Philips EM208 transmission electron microscope operated at 80 kV accelerating voltage providing samples with well-scattered NPs drops on the 300-mesh carbon-coated copper grid, and the SEM image was set down by TESCAN-Vega3 under an acceleration voltage of 30–250 and a graphite grid for depositing samples. The size distribution and zeta potential of the NPs were measured by the DLS technique using a HORIBA-SZ 100.

### Synthesis of Fe_***3***_O_4_@SiO_2_/Schiff-base/Cu(II)

Fe_3_O_4_ MNPs were synthesized through the coprecipitation method^32,38^. First, the mixture of FeCl_3_.6H_2_O (1.3 g, 4.0 mmol), FeCl_2_.4H_2_O (0.9 g, 4.5 mmol), and CTAB (1.0 g) as a surfactant was mechanically stirred in a deionized-water-containing beaker (600 cm^3^) at 80 ˚C for 0.5 h. NaOH (10%) was then added dropwise with vigorous stirring to produce a black solid product until the reaction medium reached pH 10–12. Having been heated at 60 ˚C for 2 h, the black magnetic Fe_3_O_4_ product was magnetically separated followed by washing three times with ethanol and deionized water. The core–shell Fe_3_O_4_@SiO_2_ MNPs were synthesized by a modified Stöber method^32^. Fe_3_O_4_ (0.5 g) was added to a beaker containing EtOH (50.0 cm^3^), deionized water (5.0 cm^3^), and TEOS (0.2 cm^3^, 1.0 mmol), followed by dropwise addition of NaOH 10% w (5.0 cm^3^). Having been stirred at R.T. for 0.5 h, the Fe_3_O_4_@SiO_2_ product was washed with ethanol and deionized water and dried at 80 ˚C for 10 h.

In the following, the Schiff base ligand was synthesized followed by the anchoring of the Cu metal ions. In this regard, the reaction between APTES (1.0 mmol) and salicylaldehyde (1.0 mmol) in EtOH (50.0 cm^3^) at R.T for 6 h led to the formation of the Schiff base ligand. The yellow solid product was washed with ethanol and dried in a vacuum. Afterward, the Schiff-base complex of Cu(II) was prepared through the reaction between Cu(OAc)_2_.4H_2_O (1.0 mmol) and Schiff-base ligand (2.0 mmol) in EtOH (25.0 cm^3^) under reflux conditions, which resulted in a green-color product. Finally, heating the mixture of Schiff-base complex of Cu(II) (1.0 mmol) and Fe_3_O@SiO_2_ (2.0 g) in EtOH (10.0 cm^3^) under reflux conditions contributed to the formation of Fe_3_O_4_@SiO_2_/Schiff-base/Cu(II) after 12 h. The product was separated by an external magnet, washed with ethanol and water, and dried at 80 ˚C for 6 h^34^. The melting points of Fe_3_O_4_, Fe_3_O_4_@SiO_2_, and Fe_3_O_4_@SiO_2_/Schiff-base/Cu(II) were measured up to 300 ˚C, and no alteration was observed. Also, Fe_3_O_4_@SiO_2_/Schiff-base/Cu(II) as heterogeneous magnetic nanoparticles disperses well in the organic solvents without solubility.

### Antifungal measurement of Fe_3_O_4_@SiO_2_/Schiff-base/Cu(II)

The Minimum Inhibitory Concentrations (MIC) of the six main species of *Candida* genus fungi were determined using the broth microdilution method. The inocula of tested yeast fungi species from Centraal bureau voor Schimmelcultures (CBS) and American Type Culture Collection (ATCC) including *C. dubliniensis* (C 8501), *C. krusei* (A 6258), *C. tropicalis* (A 750), *C. parapsilosis* (A 4344), *C. glabrata* (A 90,030) and *C. albicans* (C 562) strains, which were prepared from 24-h cultures. Fungal suspensions were adjusted to 0.5 McFarland standard turbidity which is a stock suspension of 1–5 × 10^6^ cells/mL. For determination of antifungal activities, serial dilutions of the Fe_3_O_4_@SiO_2_/Schiff-base/Cu(II) (0.5 to 256 μg/mL) were prepared in Roswell Park Memorial Institute (RPMI-1640) medium, in 96-well microtiter plates. Then 100 μL of the working inoculums of tested yeast fungi were added to the microtiter plates and incubated in a humid atmosphere at 32 °C for 24–48 h. The culture medium alone and culture medium with inoculums (yeast) were adjusted as blank and growth controls, respectively. It is noteworthy that each experiment was carried out in triplicate. After incubation time, the existence of growth in 96-well microtiter plates was compared with the growth control. The lowest concentration of the mentioned treatments, without visible growth, was considered MIC. Additionally, 10 μL medium from no visible growth of yeast fungi wells on Sabouraud Dextrose Agar (SDA) was applied to specify Minimum Fungicidal Concentration (MFC). The MFC values were assessed as the lowest concentrations producing no more than 4 colonies (demonstrating 99.9% mortality of the fungi in the initial inoculums)^39,40^.

### The MTT assay

The cytotoxicity of the produced Fe_3_O_4_@SiO_2_/Schiff-base/Cu(II) NPs was measured quantitatively using the MTT test^41^. At a density of 5 × 10^3^ cells per well in a 96-well cell culture plate (5000 cells/96 well), the mouse L929 murine fibroblastic cell line was grown at 37 ˚C with CO_2_ in a humidified incubator containing DMEM/F12 (1:1 mixture of Dulbecco's Modified Essential Medium (DMEM) and Ham's F-12 Medium) culture media supplemented with 10% (v/v) fetal bovine serum (FBS), 100 unit/ml of penicillin and 100 µg/ml of streptomycin. 16, 32, and 64 μg/mL of sterilized Fe_3_O_4_@SiO_2_/Schiff-base/Cu(II) MNPs were added to each row (8 replicates). No treatment was added to one row and it was considered as the control group. At each time point (1, 3, and 5 days after cells seeding), the culture medium was removed and 0.2 ml of MTT (0.5 mg/mL) was added to each well, and the cells were incubated at 37 ˚C for 4 h in an incubator in dark condition. Then, the solution was removed and 0.1 mL DMSO was added to each well to dissolve the formed formazan crystals. The absorption values (OD) of the samples were measured at a wavelength of 570 nm utilizing a microplate reader of Biotech Instruments.

## Results and discussion

### Preparation of the Fe_3_O_4_@SiO_2_/Schiff-base/Cu(II) MNPs

**Figure 1:**
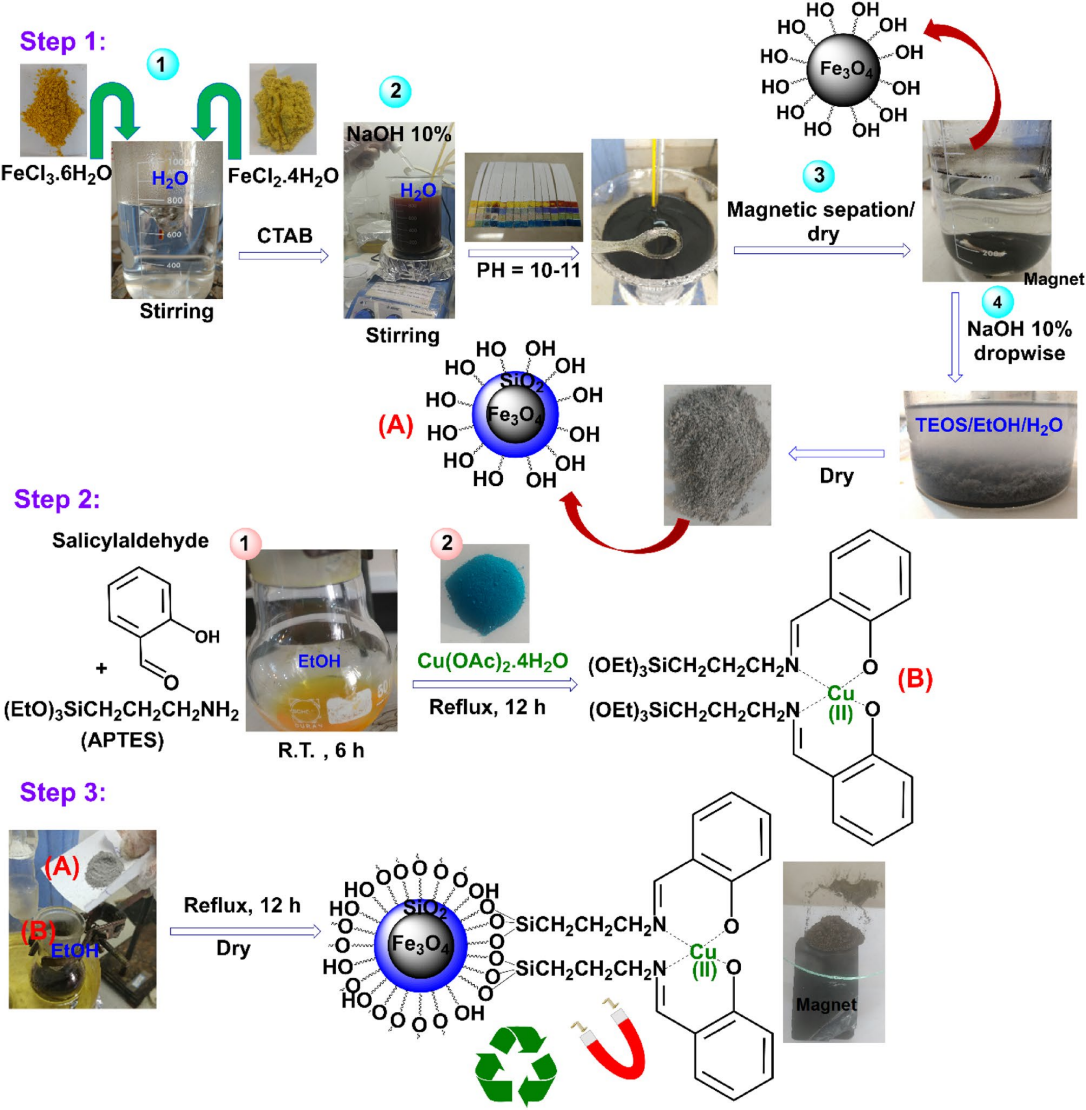
Preparation process of Fe_3_O_4_@SiO_2_/Schiff-base/Cu(II) MNPs.

As described in the experimental section, the preparation of Fe_3_O_4_@SiO_2_/Schiff-base/Cu(II) MNPs is briefly shown in Fig. 1. First, the coating of the Fe_3_O_4_ core NPs being formed by the combination of Fe (II) and Fe (III) chloride salts was done by using the silica source of TEOS under PH control to afford Fe_3_O_4_@SiO_2_ core–shell NPs. Second, the Schiff base complex of Cu(II) was made through the reaction between salicylaldehyde and APTES followed by adding Cu(OAc)_2_.4H_2_O. Finally, the Schiff base complex of Cu(II) was immobilized on the surface of Fe_3_O_4_@SiO_2_ NPs under reflux conditions to obtain the Fe_3_O_4_@SiO_2_/Schiff-base/Cu(II) MNPs (Fig. 1).

### Characterization of the Fe_3_O_4_@SiO_2_/Schiff-base/Cu(II) MNPs

#### FT-IR analysis

**Figure 2:**
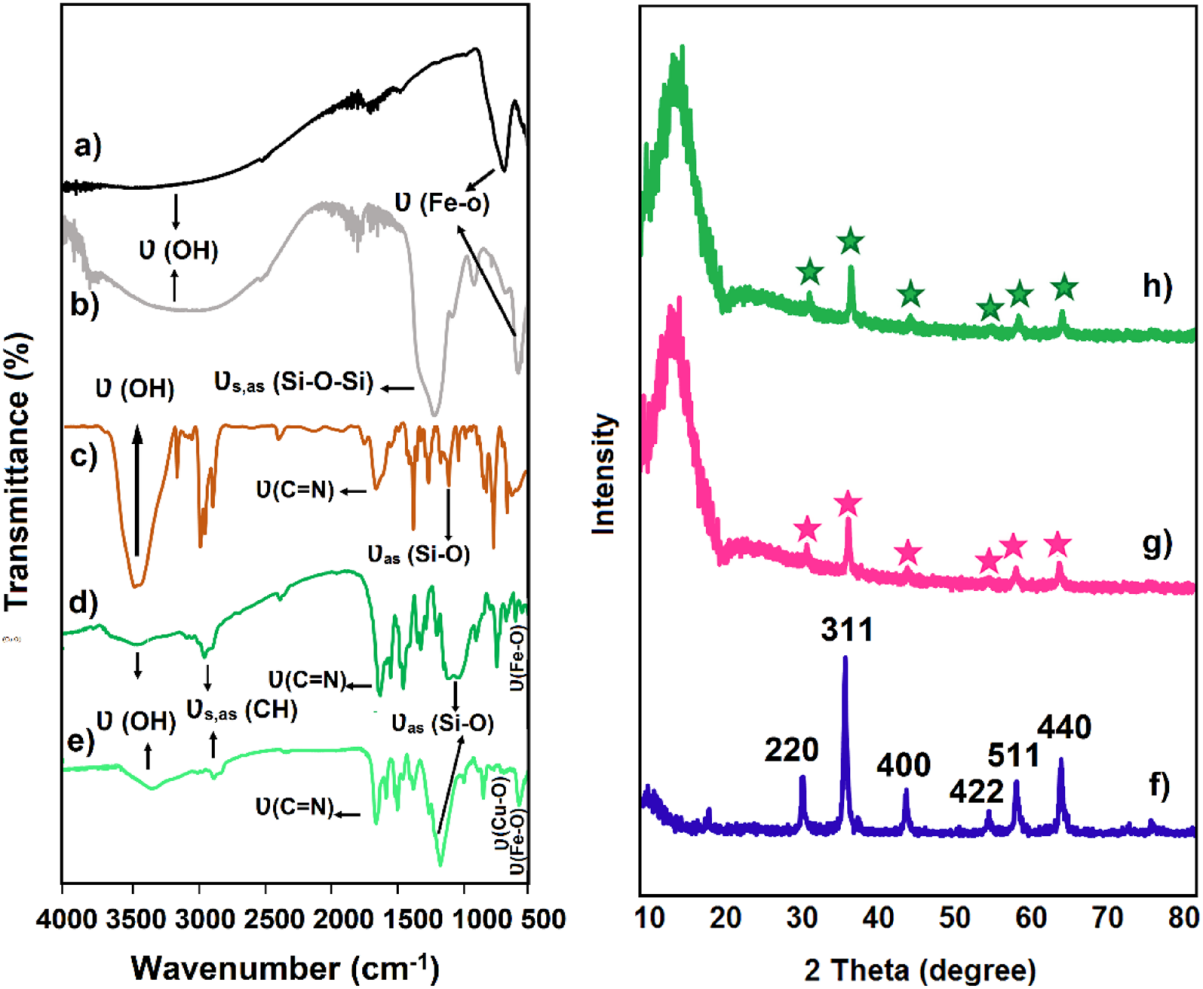
FT-IR spectra of (**a**) Fe_3_O_4_, (**b)** Fe_3_O_4_@SiO_2_, (**c**) Schiff-base ligand, (**d**) Schiff-base/Cu(II) and (**e**) Fe_3_O_4_@SiO_2_/Schiff-base/Cu(II); XRD patterns of (**f**) Fe_3_O_4_, (**g**) Fe_3_O_4_@SiO_2_, and (**h**) Fe_3_O_4_@SiO_2_/Schiff-base/Cu(II).

The FT-IR spectra of (a) Fe_3_O_4_, (b) Fe_3_O_4_@SiO_2_, (c) 2-(((3-(triethoxysilyl)propyl)imino)methyl)phenol (Schiff-base ligand), (d) Cu(II)-Schiff-base complex, and (e) Fe_3_O_4_@SiO_2_/Schiff-base/Cu(II) demonstrate the characteristic chemical bonds in the structures (Fig. 2). The Fe–O bond in the Fe_3_O_4_ and Cu–O bond in the Fe_3_O_4_@SiO_2_/Schiff-base/Cu(II) appeared at distinctive vibrational bands of 520 and 580 cm^−1^, respectively (Fig. 2e and a). Also, the Fe_3_O_4_@SiO_2_ and Fe_3_O_4_@SiO_2_/Schiff-base/Cu(II) showed the characteristic band of Si–O–Si at around 1200 cm^−1^ (Fig. 2b and e). The C = N bond in the Schiff-base ligand represented the stretching band at 1631 cm^−1^ (Fig. 2c), which appeared at the lower frequency of 1618 cm^−1^ in both the Cu(II)/Schiff-base complex (Fig. 2d) and Fe_3_O_4_@SiO_2_/Schiff-base/Cu(II) (Fig. 2e) due to the anchoring of Cu metal in the complex. Based on this observation, the synthesis of Fe_3_O_4_@SiO_2_/Schiff-base/Cu(II) has been verified by the FT-IR evidence.

#### X‑Ray diffraction analysis

**Figure 3:**
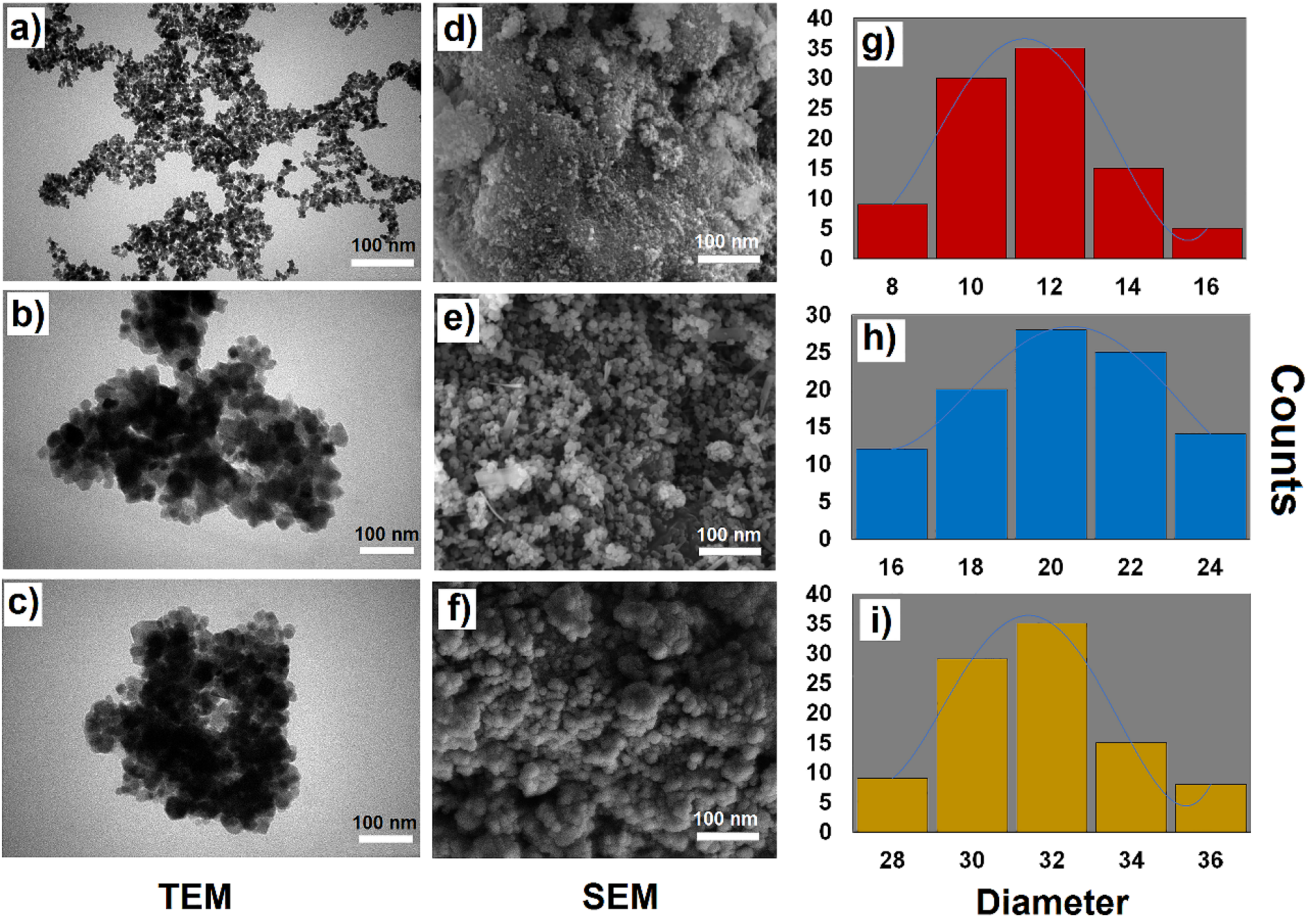
TEM images of (**a**) Fe_3_O_4_, (**b**) Fe_3_O_4_@SiO_2_ and (**c**) Fe_3_O_4_@SiO_2_/Schiff-base/Cu(II); SEM images of (**d**) Fe_3_O_4_, (**e**) Fe_3_O_4_@SiO_2_ and (**f**) Fe_3_O_4_@SiO_2_/Schiff-base/Cu(II); particle size distributions of (**g**) Fe_3_O_4_, (**h**) Fe_3_O_4_@SiO_2_, and (**i**) Fe_3_O_4_@SiO_2_/Schiff-base/Cu(II).

Figure 2f-h illustrate the XRD spectra of (f) Fe_3_O_4_, (g) Fe_3_O_4_@SiO_2_, and (h) Fe_3_O_4_@SiO_2_/Schiff-base/Cu(II). Crystallographic spinel structure in the Fe_3_O_4_ NPs is demonstrated by the six distinct patterns at 2θ = 30.5 , 35.40 , 43.16 , 56.45 , 59.2 , and 65.59 , which are associated with indices 220, 311, 400, 422, 511, and 440, respectively (Fig. 3f)^32^. The Fe_3_O_4_@SiO_2_ and Fe_3_O_4_@SiO_2_/Schiff-base/Cu(II) also contain the mentioned peaks at lower intensities (Fig. 3g,h), which outlines the successful coating of the Fe_3_O_4_ NPs with SiO_2_ and Schiff base complex of Cu. Also, the distinct diffusion peak at 2θ = 15–25  in Fe_3_O_4_@SiO_2_ results from the coating of Fe_3_O_4_ MNPs by silica (Fig. 3g)^32^, which appears at lower angles due to the binding of Cu(II)-Schiff base complex to the Fe_3_O_4_@SiO_2_ (Fig. 3h). So, the XRD outcome supports the efficient immobilization of the Cu(II)-Schiff base complex on the Fe_3_O_4_@SiO_2_ MNPs without any alteration in the structure of Fe_3_O_4_ MNPs.

### SEM, TEM, DLS results

TEM, SEM, and DLS results of Fe_3_O_4_, Fe_3_O_4_@SiO_2_, and Fe_3_O_4_@SiO_2_/Schiff-base/Cu(II) MNPs are depicted in Fig. 3. The TEM image of Fe_3_O_4_ displays the harmonic dark spheres with an estimated particle size of 10–15 nm (Fig. 3a). The grey silica layer on the surface of Fe_3_O_4_ NPs results in a TEM image with maintaining the spherical pattern, which makes the Fe_3_O_4_@SiO_2_ MNPs of 20–25 nm in size (Fig. 3b). The immobilization of Cu(II)/Schiff-base complex on the Fe_3_O_4_@SiO_2_ NPs did not affect the spherical structure pattern and an average size of 30–38 nm was obtained for the Fe_3_O_4_@SiO_2_/Schiff-base/Cu(II) MNPs (Fig. 3c). The uniform spheres and morphological patterns are shown in the NPs (Fig. 3d-f), which confirms the effective surface modification of the Fe_3_O_4_ MNPs with silica layer and the Fe_3_O_4_@SiO_2_ with Cu metal complex afterward^34^. The mean particle size distribution of Fe_3_O_4_, Fe_3_O_4_@SiO_2_, and Fe_3_O_4_@SiO_2_/Schiff-base/Cu(II) MNPs was measured by the DLS analysis, which shows the average size of 12, 20, and 32 nm, respectively (Fig. 3g-i).

### EDX spectrum

**Figure 4:**
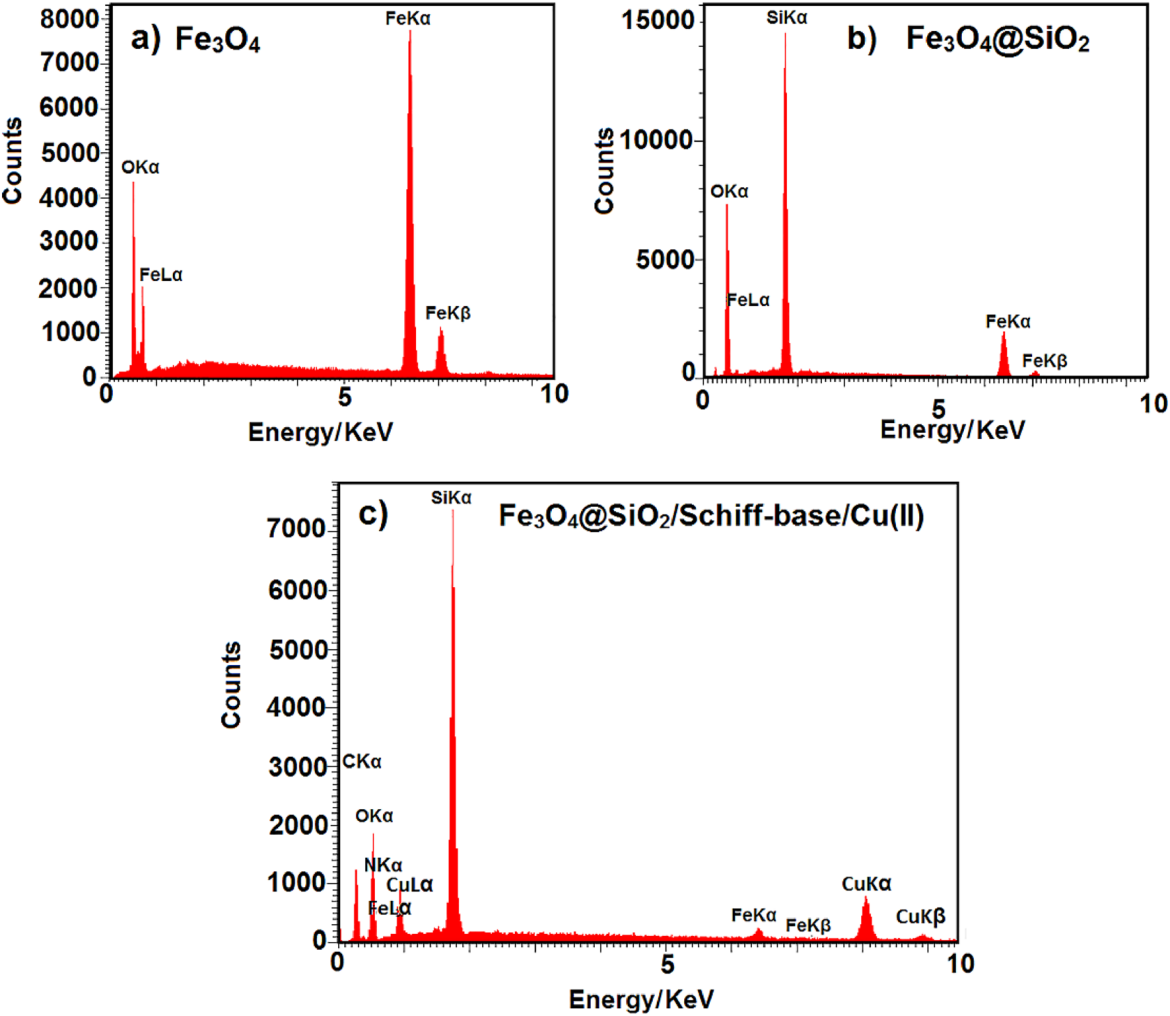
EDX spectra of (**a**) Fe_3_O_4_ (**b**) Fe_3_O_4_@SiO_2_ and (**c**) Fe_3_O_4_@SiO_2_/Schiff-base/Cu(II).

**Figure 5:**
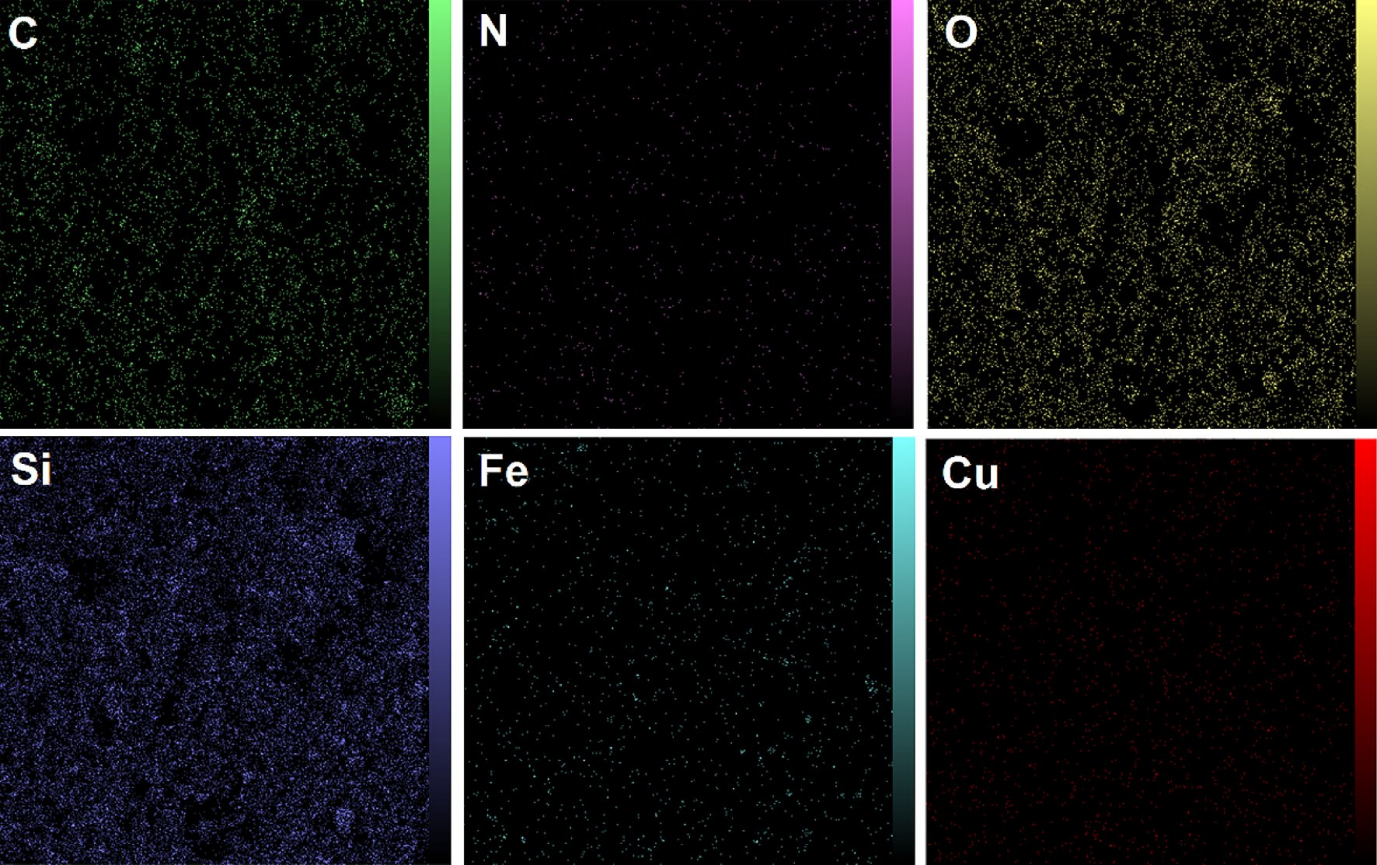
Map images of the elements (C, N, O, Si, Fe, and Cu) in Fe_3_O_4_@SiO_2_/Schiff-base/Cu(II).

The EDX analysis revealed the elemental composition in the (a) Fe_3_O_4_, (b) Fe_3_O_4_@SiO_2_ and (c) Fe_3_O_4_@SiO_2_/Schiff-base/Cu(II) (Fig. 4). The characteristic elements of Fe and O in Fe_3_O_4_ are depicted in Fig. 4a. The presence of a large amount of Si in the elemental composition spectrum of Fe_3_O_4_@SiO_2_ shows the successful coating of Fe_3_O_4_ NPs by the silica layer. (Fig. 4b)^32^. As depicted in Fig. 4c, the elemental peaks of Fe, O, C, N, Si, and Cu in the Fe_3_O_4_@SiO_2_/Schiff-base/Cu(II) indicate the efficient functionalization of Fe_3_O_4_@SiO_2_ with the Cu(II)-Schiff-base complex leading to the synthesis of Fe_3_O_4_@SiO_2_/Schiff-base/Cu(II). Also, the map analysis of the Fe_3_O_4_@SiO_2_/Schiff-base/Cu(II) is illustrated in Fig. 5, which represents the presence and distribution of the C, N, O, Si, Fe, and Cu elements in the structure.

### VSM and TGA analyses

**Figure 6:**
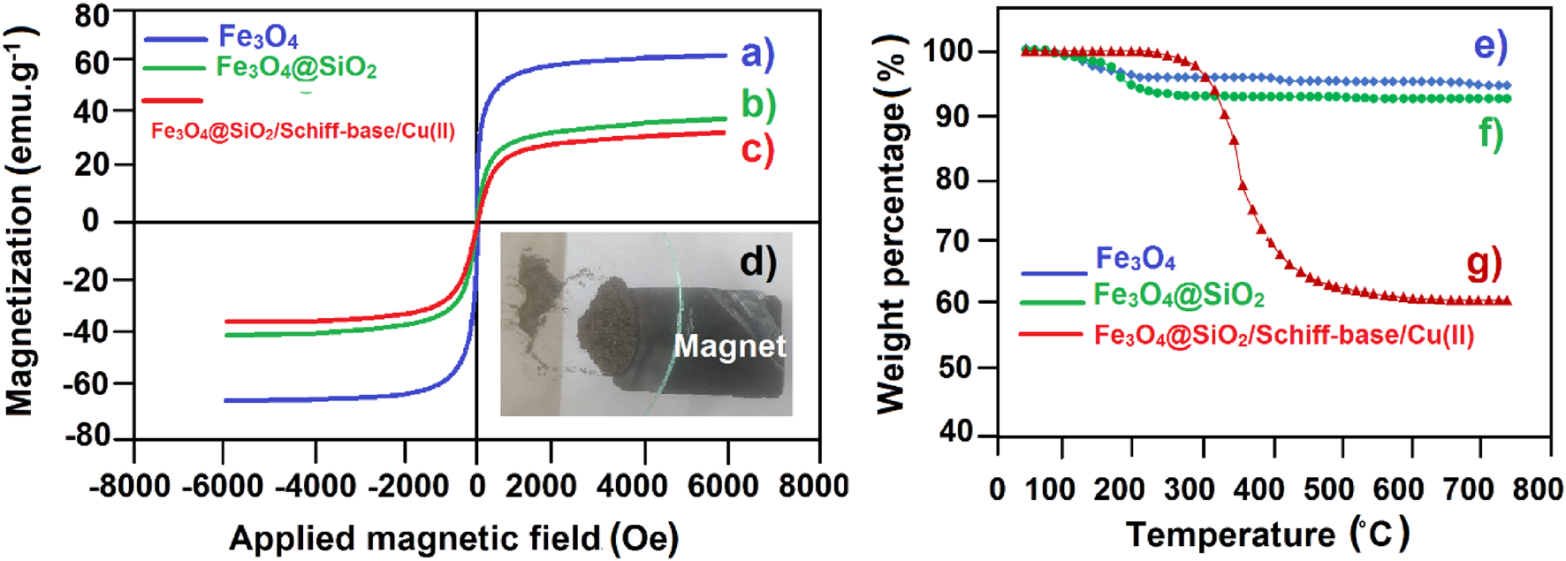
Magnetization curves of (**a**) Fe_3_O_4_, (**b**) Fe_3_O_4_@SiO_2_, and (**c**) Fe_3_O_4_@SiO_2_/Schiff-base/Cu(II), (**d**) magnetic characteristic image of Fe_3_O_4_@SiO_2_/Schiff-base/Cu(II); TGA spectra of (**e**) Fe_3_O_4_, (**f**) Fe_3_O_4_@SiO_2_ and (**g**) Fe_3_O_4_@SiO_2_/Schiff-base/Cu(II).

The superparamagnetic behavior of Fe_3_O_4_, Fe_3_O_4_@SiO_2_, and Fe_3_O_4_@SiO_2_/Schiff-base/Cu(II) MNPs was studied by VSM analysis at room temperature. A magnetic field up to 8000 Oe at 300 K was applied to investigate the super-paramagnetization (Fig. 6a-c). The magnetization feature of the NPs is confirmed by observing no hysteresis phenomenon in the magnetization curves. The saturation magnetization (Ms) values of Fe_3_O_4_, Fe_3_O_4_@SiO_2_, and Fe_3_O_4_@SiO_2_/Schiff-base/Cu(II) MNPs were found to be 68 (Fig. 6a), 45 (Fig. 6b), 34 (Fig. 6c) emu/g, respectively. These slight decreases in the Ms values of NPs stem from the functionalization of Fe_3_O_4_ with SiO_2_ core–shell and Cu(II)-Schiff base complex, subsequently. However, it does not have an influential effect on the magnetization identity of NPs, especially Fe_3_O_4_@SiO_2_/Schiff-base/Cu(II)^33^. This evidence is finally confirmed by evaluating the magnetization of NPs with an external magnetic field (Fig. 6d). The Fe_3_O_4_@SiO_2_/Schiff-base/Cu(II) would be separated simply and rapidly, which is an essential factor in retrieving the MNPs.

The thermal stability of Fe_3_O_4_, Fe_3_O_4_@SiO_2_, and Fe_3_O_4_@SiO_2_/Schiff-base/Cu(II) MNPs was analyzed by TGA in the temperature range from 25 to 750 °C (Fig. 6e-g). The thermograms show the fraction of volatile components by increasing the temperature. Negligible mass loss is observed in the Fe_3_O_4_ and Fe_3_O_4_@SiO_2_ slopes until higher temperatures reveal the thermally stable structure of the NPs (Fig. 6e,f). The removal of adsorbed water, intact organic solvents, and hydroxyl groups in the range of 100 °C and 500 °C contributes to this slight decrease^32,37^. Also, a decrease in the slope appears in the Fe_3_O_4_@SiO_2_/Schiff-base/Cu(II) MNPs until 300–350 °C, after which the mass loss is attributed to the decomposition of organic compounds (Fig. 6g). This observation verifies both the successful immobilization of Cu(II)/Schiff-base complex on the Fe_3_O_4_@SiO_2_ MNPs and the excellent thermal resistance of the Fe_3_O_4_@SiO_2_/Schiff-base/Cu(II) MNPs at high temperatures.

### Zeta potential of Fe_3_O_4_@SiO_2_/Schiff-base/Cu(II)

**Figure 7:**
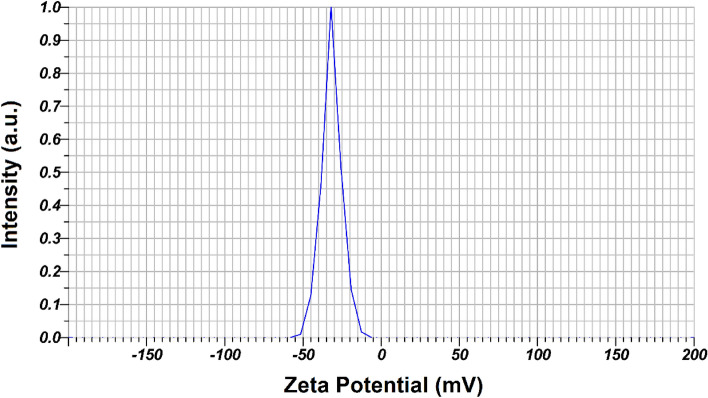
Zeta potential of Fe_3_O_4_@SiO_2_/Schiff-base/Cu(II) MNPs.

According to Fig. 7, the zeta potential magnitude of Fe_3_O_4_@SiO_2_/Schiff-base/Cu(II) MNPs obtained −31.8 mV, which indicates the stability of colloidal dispersions and the surface charge. The value of -31.8 Mv indicates the moderate to good stability of the Cu MNPs and the negative charge on the surface of Cu MNPs. This major factor contributed to the stability of Fe_3_O_4_@SiO_2_/Schiff-base/Cu(II) MNPs in suspension, and successful initial adsorption onto the cell membrane of fungi infections.

### Antifungal activity of Fe_3_O_4_@SiO_2_/Schiff-base/Cu(II)

Table 1 represents the MIC and MFC values of the Fe_3_O_4_@SiO_2_/Schiff-base/Cu(II) towards the studied fungal species. In this study, the antifungal activity of Fe_3_O_4_@SiO_2_/Schiff-base/Cu(II) was investigated against 6 *Candida* species. The Fe_3_O_4_@SiO_2_/Schiff-base/Cu(II) inhibited the growth of all of the examined yeast strains at concentrations of 8–64 μg/mL. Throughout the study, *C. parapsilosis* had the lowest MIC and MFC, which were 8 and 16 µg/mL, respectively while *C. krusei* exhibited the highest MIC and MFC (64, 256 µg/mL). As a result, *C. parapsilosis* was the most susceptible to Fe_3_O_4_@SiO_2_/Schiff-base/Cu(II). In simpler words, the Fe_3_O_4_@SiO_2_/Schiff-base complex of Cu(II) is more effective in inhibiting or killing *C. parapsilosis*.

**Table 1:** Antifungal effects of Fe_3_O_4_@SiO_2_/Schiff-base/Cu(II) on the fungi strains based on broth microdilution method.

Fungi Standard strains	Organisms	ATCC/CBS	Fe_3_O_4_@SiO_2_/Schiff-base/Cu(II)		Fluconazole
			MIC_90_ (μg/mL)	MFC (μg/mL)	MIC (μg/mL)
Fungi species	*C. dubliniensis*	C (8501)	32	128	2
	*C. krusei*	A (6258)	64	256	64
	*C. tropicalis*	A (750)	32	64	32
	*C. parapsilosis*	A (4344)	8	16	2
	*C. glabrata*	A (90,030)	16	32	32
	*C. albicans*	C (562)	32	32	8

This efficacy might stem from the small particle size of the Cu NPs, leading to a gradual release of the therapeutic agent. Although fluconazole as a standard drug, shows higher effectiveness in some instances, as indicated by MIC values, the slow-release characteristic of Fe_3_O_4_@SiO_2_/Schiff-base complex of Cu(II), along with a reduced propensity to cause drug resistance, could render it a more viable option for long-term treatment. This aspect is especially crucial for treating persistent fungal infections and in scenarios where resistance to fluconazole is a concern, potentially enhancing therapeutic effectiveness.

**Figure 8:**
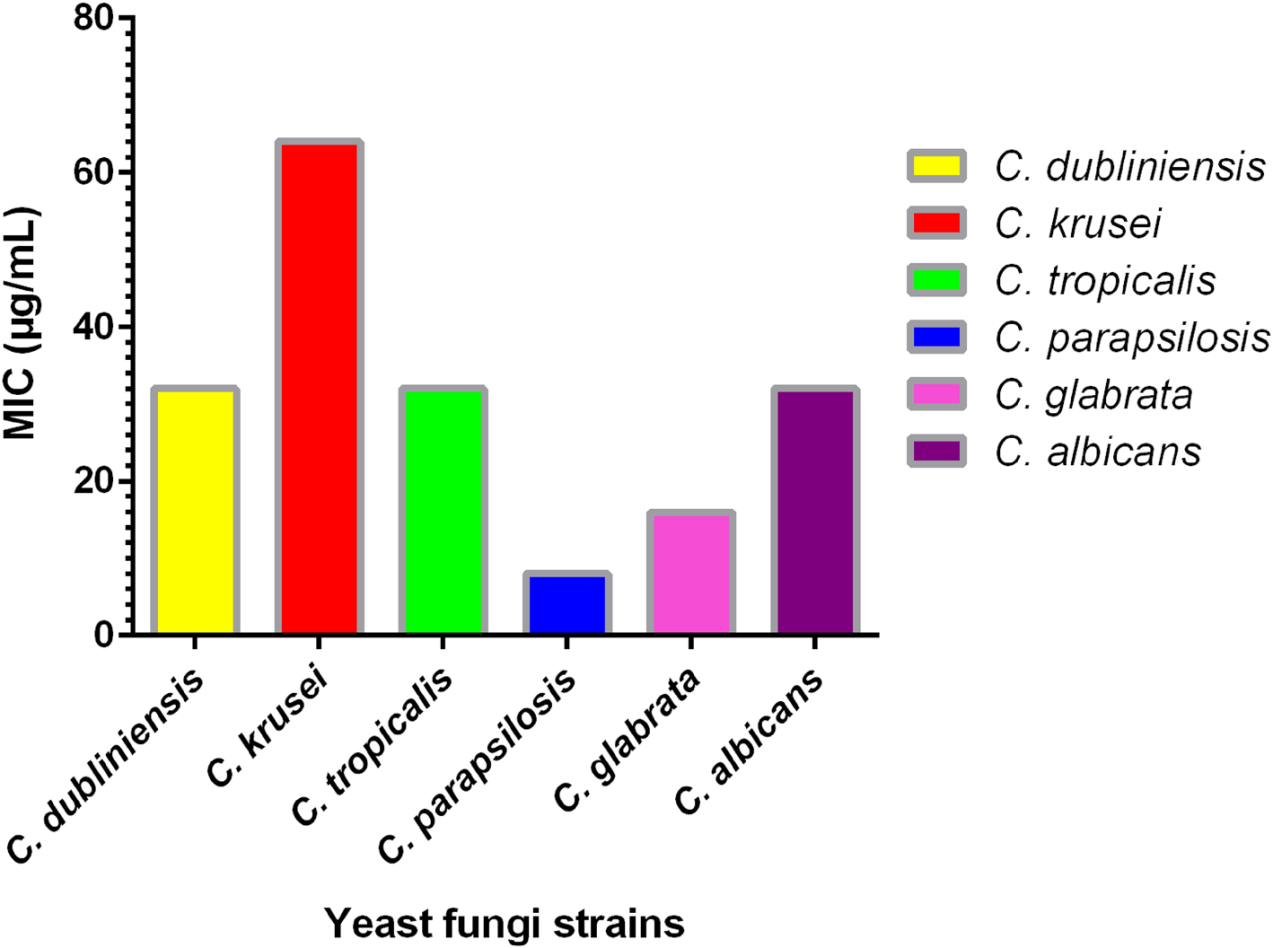
The MIC amounts of Fe_3_O_4_@SiO_2_/Schiff-base/Cu(II) MNPs treating with *Candida* species.

As depicted in Fig. 8, *C. parapsilosis* placed first in treating with the Fe_3_O_4_@SiO_2_/Schiff-base/Cu(II) MNPs with the lowest amount of 8 μg/mL. *C. glabrata* ranked the second position with the amount of 16 μg/mL of Fe_3_O_4_@SiO_2_/Schiff-base/Cu(II) MNPs. *C. albicans, C. tropicalis* and *C. dubliniensis* held the subsequent positions with the same amounts of antifungal agent (32 μg/mL). *C. krusei* occupied the last position, for which 64 μg/mL of the Fe_3_O_4_@SiO_2_/Schiff-base/Cu(II) MNPs for inhibition was used.

*C. albicans* is a common cause of candidemia, and its resistance to some antifungal drugs such as fluconazole can limit treatment options. It can cause a wide range of clinical manifestations and easily spread through the hands of healthcare workers, leading to nosocomial infections^42^. To address this problem, the obtained result reveals the potential of Fe_3_O_4_@SiO_2_/Schiff-base/Cu(II) can be an efficient candidate based on the obtained results. Therefore, regarding *C. albicans* as the most frequent fungus causing a range of infections from mild skin and mucosal infections to life-threatening invasive infections^43^, the Fe_3_O_4_@SiO_2_/Schiff-base/Cu(II) showed satisfactory antifungal activity.

Significantly, the creation of the Fe_3_O_4_@SiO_2_@Schiff-base/Cu (II) MNPs has led to an innovative approach in controlled drug release and precise drug targeting for localized fungal infections, including fungal endocarditis. This is achieved through the application of an alternating current magnetic field. The magnetic characteristics of Fe_3_O_4_@SiO_2_@Schiff-base/Cu (II) might enhance the accurate delivery of the therapeutic agent to the affected tissues. This can be accomplished using an external magnetic field, either on its own or in conjunction with other medicinal drugs^44^.

### Antifungal mechanism of Fe_3_O_4_@SiO_2_/Schiff-base/Cu(II) MNPs

**Figure 9:**
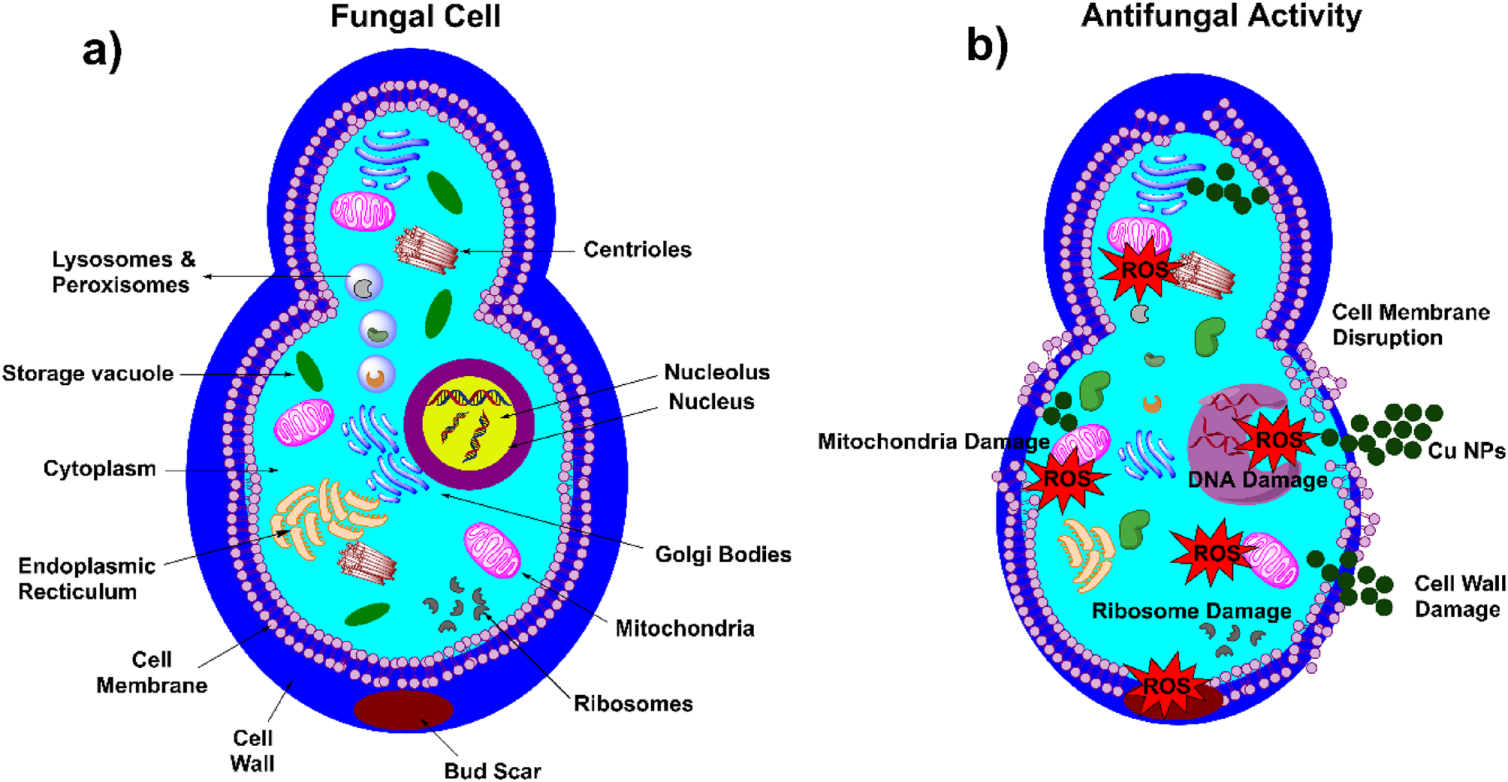
(**a**) Fungal cell structure, (**b**) antifungal activity of Fe_3_O_4_@SiO_2_/Schiff-base/Cu(II) MNPs and plausible disruption of cell structure.

The fungal cell constitutes a wide range of substructures, in which the cell membrane, cell wall, ribosomes, mitochondria, storage vacuoles, Golgi bodies, and DNA are vital organelles (Fig. 9a)^45^. The hypothetical antifungal mechanism of Cu-NPs is depicted in Fig. 9b. Although the antifungal activities of different Cu-NPs have been studied, the precise mechanism of action for NPs is not yet recognized. Based on previous reports, the cell wall of fungi can be destroyed through protein denaturation by confronting Cu-NPs, which occurs through interaction between the surface of fungi covered with carboxyl, amino, and sulfhydryl groups in the peptidoglycan layer and Cu-NPs^46^. Having been penetrated to the cell wall by endocytosis, the Cu-NPs damage the cell membrane leading to decreasing the electrochemical potential and destroying the integrity, subsequently^46^. Releasing the Cu ions in fungi cells is followed by the generation of ROS. The ROS paves the way for DNA fragmentation, enzyme activity inhibition, ribosome and protein denaturation, mitochondria damage, and affecting other components, which ends in cell death, consequently^47^.

It is noteworthy that the antifungal activity of Fe_3_O_4_@SiO_2_/Schiff-base/Cu(II) against *Candida* species can be attributed to several factors. Firstly, the presence of Cu(II) ions in the composite material can disrupt the cell membrane of *Candida* species, leading to cell death. Secondly, the Schiff base ligand in the composite material can be able to block a specific metabolic pathway that is essential for the microorganism's survival and enhance its antifungal activity, subsequently. Additionally, the Fe_3_O_4_@SiO_2_ core–shell structure provides a large surface area for interaction with *Candida* cells, increasing the effectiveness of the antifungal activity of this product^48^. Besides, the magnetic properties of Fe_3_O_4_ allow to use of a magnetic field by which the drug can be directed to the site of infection, and a lower dose of the drug sets the stage for improving the therapeutic efficacy and reducing toxicity^39^.

### Cell viability and proliferation assessment

**Figure 10:**
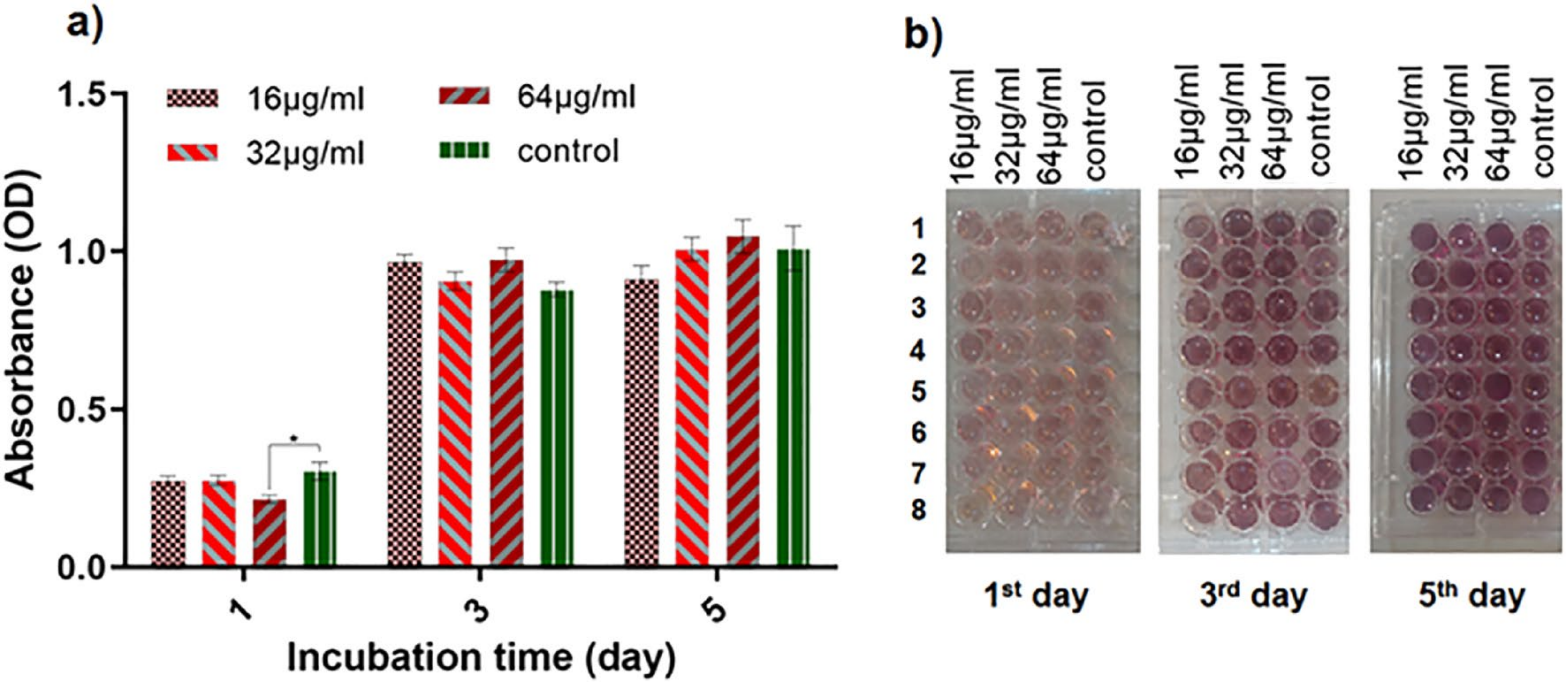
Cell viability (MTT assay): (**a**) the proliferation of control and different concentrations of samples during the incubation times of the first, third, and fifth day, (**b**) representation of cell plates with different concentrations.

An MTT assay was carried out to further explore the L929 cell proliferation with Fe_3_O_4_@SiO_2_/Schiff-base/Cu(II) MNPs on days 1, 3, and 5. As shown in Fig. 10a, a steady upward trend can be observed for 32- and 64-μg/mL samples over time. The cell viability in the 16-μg/mL sample increased on the two first days despite a minor decrease observed on the 5th day. The only significant difference between the groups was observed on the first day when the OD value of 64-μg/mL sample was less than the control group. Based on these findings, the results of the cell proliferation assay revealed that the incorporation of Cu within NPs not only had no cytotoxic effect on cells but triggered the growth of cells containing the Fe_3_O_4_@SiO_2_/Schiff-base/Cu(II) MNPs in almost all days as well as the control group. Figure 10b indicates a schematic view of the MTT test and cell growth observation over a specific time.

## Conclusion

Herein, we successfully prepared the Schiff-base ligand of Cu(II) supported on the Fe_3_O_4_@SiO_2_ MNPs using the co-precipitation method and simple immobilization. The characterization results confirmed the specific absorption peaks, the maintenance of the spinel crystalline pattern, magnetic behavior, thermal stability, nano-sized and morphological structure of the Fe_3_O_4_@SiO_2_/Schiff-base/Cu(II) MNPs by FT-IR, XRD, VSM, TGA, TEM, SEM and DLS techniques. The striking feature of the study aimed at the antifungal ability of Fe_3_O_4_@SiO_2_/Schiff-base/Cu(II) MNPs against *C. dubliniensis*, *C. krusei*, *C. tropicalis*, *C. parapsilosis*, *C. glabrata* and *C. albicans* strains. The microdilution method using 0.5–256 μg/mL dilutions of Fe_3_O_4_@SiO_2_/Schiff-base/Cu(II) MNPs as antifungal agent revealed the lowest inhibition and fungicidal concentrations at 8 and 16 µg/ml for *C. parapsilosis.* The opposite result was recorded for *C. krusei* with the highest MIC and MFC (64, 256 µg/mL). The growth of *C. Albicans* as the leading cause of fatal infections was inhibited at the concentration of 32 µg/ml of Fe_3_O_4_@SiO_2_/Schiff-base/Cu(II) MNPs. Also, the cytotoxicity of Fe_3_O_4_@SiO_2_/Schiff-base/Cu(II) MNPs was examined on the mouse L929 murine fibroblastic cell, which showed no toxicity by MTT test. That is why we can identify the Fe_3_O_4_@SiO_2_/Schiff-base/Cu(II) MNPs as an efficient antifungal candidate to address the infectious issues arising from fungal strains.

## Data Availability

All data generated or analyzed during this study are included in this published article.
